# Clinical Trials with Combination of Cytokine-Induced Killer Cells and Dendritic Cells for Cancer Therapy

**DOI:** 10.3390/ijms20174307

**Published:** 2019-09-03

**Authors:** Francesca Garofano, Maria A. Gonzalez-Carmona, Dirk Skowasch, Roland Schmidt-Wolf, Alina Abramian, Stefan Hauser, Christian P. Strassburg, Ingo G. H. Schmidt-Wolf

**Affiliations:** 1 Department of Integrated Oncology, Center for Integrated Oncology (CIO), University Hospital Bonn, Venusberg-Campus 1, 53127 Bonn, Germany; 2 Department of Internal Medicine I, University Hospital Bonn, 53127 Bonn, Germany; 3 Department of Internal Medicine II, University Hospital Bonn, 53127 Bonn, Germany; 4 Department of Anesthesiology and Postoperative Intensive Care Medicine, University Hospital Bonn, 53127 Bonn, Germany; 5 Department of Obstetrics and Gynecology (Senology), University Hospital Bonn, 53127 Bonn, Germany; 6 Department of Urology, University Hospital Bonn, 53127 Bonn, Germany

**Keywords:** adoptive cellular immunotherapy, Cytokine-induced killer cells, Dendritic cells

## Abstract

Adoptive cellular immunotherapy (ACI) is a promising treatment for a number of cancers. Cytokine-induced killer cells (CIKs) are considered to be major cytotoxic immunologic effector cells. Usually cancer cells are able to suppress antitumor responses by secreting immunosuppressive factors. CIKs have significant antitumor activity and are capable of eradicating tumors with few side effects. They are a very encouraging cell population used against hematological and solid tumors, with an inexpensive expansion protocol which could yield to superior clinical outcome in clinical trials employing adoptive cellular therapy combination. In the last decade, clinical protocols have been modified by enriching lymphocytes with CIK cells. They are a subpopulation of lymphocytes characterized by the expression of CD3+ and CD56+ wich are surface markers common to T lymphocytes and natural killer NK cells. CIK cells are mainly used in two diseases: in hematological patients who suffer relapse after allogeneic transplantation and in patients with hepatic carcinoma after surgical ablation to eliminate residual tumor cells. Dendritic cells DCs could play a pivotal role in enhancing the antitumor efficacy of CIKs.

## 1. Introduction

Cytokine induced killer cells (CIKs) are a heterogeneous population of polyclonal T lymphocytes obtained via ex vivo expansion of lymphocytes. They share phenotypic and functional characteristics with both, T cells and NK cells. Initially described by Schmidt-Wolf et al. in 1991 [1], CIKs are efficiently expanded in vitro by incubation of peripheral blood mononuclear cell PBMCs with the timely addition of IFN-γ (1000 IU/mL) on day 0 of culture, mAb anti-CD3 (OKT3) (50 ng/mL) and IL-2 (500 IU/mL) on the next day, followed by the subsequent addition of IL-2 during culture [2]. They possess a high proliferation rate and potent antitumor effects. They are capable of exerting a potent MHC-unrestricted cytotoxicity against both hematologic and solid tumors, but not hematopoietic precursors and normal tissues. Within the heterogeneous T cell population two main subpopulations can be distinguished, one coexpressing the CD3 and CD56 molecules (range: 40% to 80%), while the other presenting a CD3+ CD56- phenotype (range: 20% to 60%). It also comprises a small subpopulation (<10%) of CD3- CD56+ NK cells. The antitumor efficacy of CIKs has been reported to be associated with the CD3+ CD56+ subset [2,3]. The addition of IFN-γ during generation of CIKs activates monocytes providing them with a contact-dependent factor CD58 (lymphocyte function associated antigen-3 LFA-3) and a soluble factor IL-12. These two factors are important for the expansion to CD56-positive T cells and the acquisition of T helper 1 phenotype of CIK cells [4]. CIKs are able to secrete TNF-α, IL-2 and IL-6 but are not able to secrete IL-4, IL-7 and IL-12. Morphologically, CIKs are large and completely granulated. They cannot be distinguished from NK cells. The complementary subsequent addition of anti-CD3 acts as a mitogenic stimulus and high doses of IL-2 principally promote the expression of the natural killer group 2 member D (NKG2D) transmembrane adapter protein DAP10, which in turn is essential for cytolysis [5]. Different strategies have been developed in order to improve the proliferation and efficacy of CIK cells by the addition of other soluble factors. IL-15 plays an important role in the immune system. It is also able to further activate CIK cells [6]. A significantly increased anti-leukemic activity of CIK cells stimulated with the IL-15 modified protocol was observed compared to the conventional IL-2-activated CIK cells [7]. IL-21 added to the cell culture increases anti-leukemic activity by enhancing the expression of perforin, granzyme B, FasL, IFN-γ and TNF-α [8]. The use of CIKs clinically is widely facilitated by the reproducibility and simplicity of the expansion protocol and their significant MHC-independent antitumor efficacy against a broad range of cancers. Compared to lymphokine-activated killer (LAK) cells which are induced by incubation with interleukin (IL) and tumor-infiltrating lymphocytes (TILs), CIK cells can be obtained more easily and reveal a higher tumor-specific cytotoxic activity. Althought they are missing the Fcγ receptor CD16, which is a mediator of Antibody-dependent cell-mediated cytotoxicity ADCC mechanisms, it was observed in 2016 CIK cells to possess a donor dependent expression of CD16 which can have a strong effect both in vitro and in vivo [9]. However, a few groups did not observe any expression of this Fcγ receptor [10] leading to some disagreement [11].

More extensive phenotypic analysis of expanded CD3+CD56+ cells showed that the most part express the T-cell receptor TCR αβ and are CD8+, but CD4+ cells can also be found to a lesser extent within CIKs cultures. Other phenotypic characteristics of CIKs are the expression of CD5, CD11b and CD57 molecules and the Human Leukocyte Antigen—antigen D Related (HLA-DR) an MHC class II cell surface receptor. Unlike classic NKT cells, CIKs express a polyclonal TCR repertoire and are also independent from CD1 for their expansion. As the most important antigen presenting-cells (APCs), dendritic cells DCs are professional antigen-presenting cells which have the particular ability to stimulate both innate and adaptive antitumor immune responses. DCs, in considerable numbers, can be derived in vitro from both CD14+ cells (monocytes) and from CD34+ cells (hematopoietic progenitor stem cells) using appropriate cytokine cocktails directly from the blood in order to arm them with specific antigens. In the recent years, DCs and CIKs have been used in combination for cancer immunotherapy. Studies have shown that contact interactions between CIKs and DCs led to changes in the expression of the surface molecules of both populations and to a significantly high increase of IL-12 secretion which was independent of binding on the IL-12 receptor. Coculturing CIK cells with DCs eventually resulted in a higher cytotoxic activity of CIKs and also led to cytotoxic activity against a tumor cell line that was resistant to CIKs without coculture [12]. The use of antigen-pulsed autologous dendritic cells to stimulate cytotoxic T-cell anti-tumor immune responses has been developed in human clinical trials involving cancer. The procedure for DC-CIK cells preparation and infusion is the isolation of PBMCs collected from patients or healthy donors and the DC-CIK cells are generated ex-vivo. They are harvested and analyzed for phenotype, then suspended in saline for intravenous injection. 

Immunotherapy with immune checkpoint inhibition has been a therapy showing success in cancer treatment. For the purpose to differentiate between self and non-self the immune system depends on different “checkpoints”. They are molecules present in certain immune cells and need to be activated or inactivated to start an immune response. Often tumor cells are able to avoid detection by the immune system both systematically and locally. Examples of checkpoint inhibitors are represented by the molecules PD-1 and PD-L1. PD-1 is expressed on DCs, monocytes, activated T cells, B cells and NK cells resulting in immune suppression and enhancement of immune surveillance. PDL-1 is expressed on solid tumors, tumor-infiltrating DCs and macrophages. PDL-1 also interacts with PD-1.

### 1.1. Activation Receptors: NKG2D, DNAM-1, CD56 and NKp30

**Figure 1:**
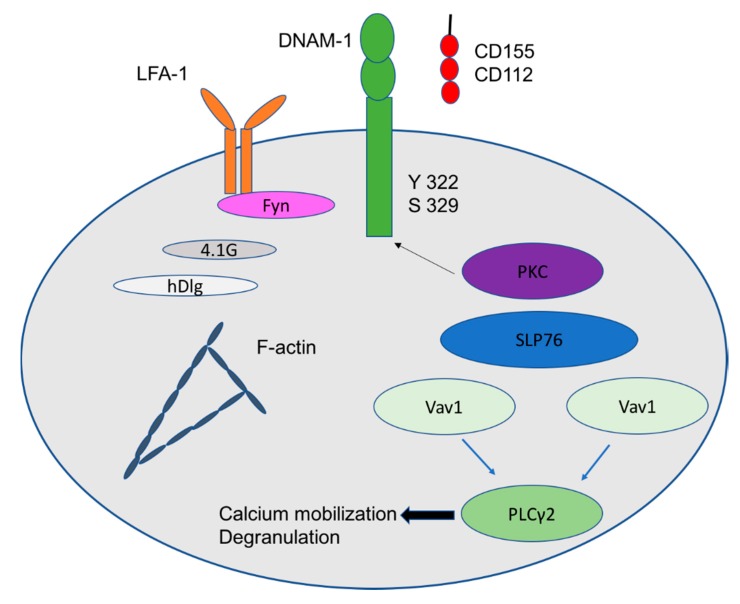
Signaling pathway of DNAM-1 receptor.

NKG2D receptor, a type-2 transmembrane homodimer, in association with DAP10 results in tyrosine phosphorylation on the immunoreceptor tyrosine-based activating motif YINM motif. This leads to recruitment of the phosphoinositide 3-kinase PI3K and a cascade of growth factor receptor-bound protein 2 Grb2. On CD8 T cells NKG2D is able to act as a co-stimulatory receptor, requiring T cell receptor TCR-mediated signaling for its activation [13]. The interaction between the NKG2D receptor and its ligands (e.g., MICA, MICB, and ULBP 1–4) mediates the release of granzyme B and perforin leading to perforin-mediated tumor cell lysis. NKG2D ligands are overexpressed on both solid and hematologic tumors making them attractive to CIK cell-induced cytolysis [14,15,16]. In addition to cytotoxicity, it promotes the production of different cytokines, including interferon IFN-γ and tumor necrosis factor TNF-α. DNAX accessory molecule (DNAM-1, CD226), member of the family of receptors that recognize nectin and nectin-like molecules, is an adhesion molecule which is a critical regulator of the cytotoxicity of NK cells upon interaction with its ligands CD155 and CD112. Its expression is regulated by cellular stress in different pathological conditions [17]. After engagement with its ligands, DNAM-1 relocates to lipid rafts and binds to actin cytoskeleton through its association with 4.1G, member of the 4.1 family of proteins, and human discs large protein (hDLG)/SAP97, member of the membrane-associated guanylate kinase (MAGUK) family of proteins. In addition, the intracellular domain of DNAM-1 gets phosphorylated in Ser329 by proteinkinase C [18]. This causes the association of DNAM-1 with the lymphocyte function-associated antigen 1 *LFA*-*1* which is an integrin. In turn, LFA-1 recruits the Fyn Src kinase in order to phosphorylate the Tyr322 of DNAM-1 intracellular domain. This initiates the downstream signalling leading to lymphocyte cytosolic protein two LCP2, also known as *SLP*-*76*, a signal-transducing adaptor protein inhibition (phosphorylation) of Vav1. It also leads to Phospholipase Cγ2 (PLCγ2) activation allowing calcium burst and degranulation (Figure 1). Another archetypal phenotypic marker of NK cell is represented by CD56, also called neural cell adhesion molecule (NCAM). It is a homophilic binding glycoprotein, a member of the immunoglobulin superfamily, which has a role in CIK-mediated lysis against CD56 positive hematopoietic cells. Blocking of CD56 with the anti-CD56 monoclonal antibody and knockdown of CD56 in CIK cells by short interfering RNA (siRNA) significantly reduced CIK-mediated lysis of three CD56^+^ hematopoietic tumor cell lines [19].

NKp30 receptor also called CD337 is expressed in low levels on CIK cells and has been shown to be involved in the reciprocal activation between CIK and dendritic cells DC [12]. CIK cells can efficiently kill immature DC and secrete IFN-γ in response to immature DCs by perforin mediated mechanism.

### 1.2. Inhibitory Receptor

Immunotherapy with immune checkpoint inhibition has been successful in cancer treatment. For the purpose of differentiating between self and non-self the immune system depends on different “checkpoints”. Checkpoints are molecules present in certain immune cells and need to be activated or inactivated to start an immune response. Often tumor cells are able to avoid detection by the immune system both systematically and locally. The use of immune checkpoint inhibitors with CIK cells may be a promising strategy to achieve a higher clinical outcome. Programmed death ligand 1 or PD-L1 is one of the inhibitory ligands that, in many cancers, can help tumor cells evade the immune system when overexpressed. PD-L1 interferes with the antitumor immune response by binding to its receptors B7.1 and PD-1, deactivating T cells cytotoxic activity. CIK cells express PD-1 on the cell surface. A recent article evaluated the effect of PD-1/PD-L1 pathway blockade on the CIK cytotoxic activity [20]. This study demonstrated an enhancement of the CIK cell cytotoxic activity by release of IFN-γ, increase in NKGD2 receptor levels and expression of CD107a.

### 1.3. Fas-Mediated Apoptosis

**Figure 2:**
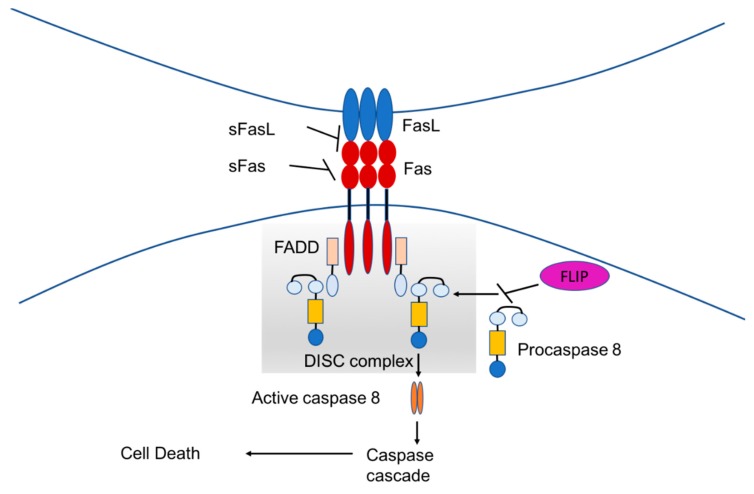
Schematic representation of the Fas/Fas-L pathway.

The Fas (also called CD95 or APO-1 or TNFRSF6)/Fas-L pathway, which results in apoptotic cell death, is a mechanism mediated by caspase activation. The activation of this pathway is regulated by different mechanisms, including formation of Fas microclusters and actin reorganization. During an immune response against cancer cells, the immune effector cells can express both Fas and FasL. Target tumor cells can express FasL leading them to apoptosis [21,22]. Fas is a type I transmembrane protein which contains in its cytoplasmic region a death domain (DD), essential for the induction of apoptosis. Binding of the Fas with its ligand (FasL), a 40-kDa membrane protein, leads to recruitment of the adaptor protein Fas-associated death domain (FADD) and activation by FADD of procaspase-8, resulting in the formation of the death-inducing signaling complex (DISC). Caspase-8 directly activates caspase-3 and initiates the caspase cascade. This ultimately leads to cell death (Figure 2). Different proteins can inhibit that pathway. Anti-apoptotic proteins, which are soluble Fas and soluble FasL, bind to the respective ligands. Flice inhibitor protein FLIP inhibits the activation of caspase-8 and is a major anti-apoptotic protein. It has been shown that CIK cells were resistant to Fas-mediated apoptosis that could be induced by the expression of FasL on cancer cells [23]. However, in another work it has been shown that CIK cells are sensitive to Fas-mediated apoptosis and it could influence their cytotoxic activity in vitro [24].

In the following sections, clinical studies applying CIKs and DCs combination for the treatment of different tumors are reviewed and discussed in more detail.

## 2. Gastrointestinal Tumors

### 2.1. Hepatocellular Carcinoma

Hepatocellular carcinoma (HCC) is the fifth most common type of cancer worldwide and shows an increasing incidence. The prognosis of HCC is still very poor. Due to a diagnosis in an advanced stage in the majority of patients or due to the presence of advanced liver cirrhosis, curative options for patients are reduced to 30% of patients at the time of diagnosis. The majority of cases (more than 80%) occurred in Asia [25]. In China it is the second most common cause of cancer death and is related to infections with hepatitis B virus (HBV). In the western countries, alcoholic liver cirrhosis, chronic hepatitis C virus infection (HCV) and non alcoholic steatohepatitis are the major risk factors for the development of liver cirrhosis and HCC. Surgical treatment and liver transplantation are potentially curative treatments of HCC. However, only around 20–30% of HCC patients can undergo surgical therapy. For unresectable locally advanced HCC, the transcatheter arterial chemoembolization (TACE) alone or in combination with radiofrequency ablation RFA showing a survival benefit is widely adopted [26]. Targeted therapy with multikinase inhibitors, such as sorafenib [27], regorafenib [28], lenvantinib [29], and more recently cabozantinib [30] and with antiangiogenesis agents, such as ramucirumab [31] have shown antitumoral effects and clinical benefit towards advanced HCC. In the last two years immunotherapy with immune check point inhibitors targeting the PD-1 receptor, such as nivolumab and pembrolizumab has shown promising results in the treatment of HCC in first phase I/II clinical trials [32,33].

### 2.2. Immunotherapy with CIK Alone or in Combination with DC as Adjuvant Therapy After Resection or RFA

In 2000 Takayama et al. [34] published a study investigating the postsurgical recurrence rates of HCC. They did a randomized trial, between 1992 and 1995, in which 150 patients who had undergone curative resection for HCC were divided into two groups: one group (*n* = 76) was assigned adjuvant cytokine-stimulated lymphocyte immunotherapy; the other group (*n* = 74) received no adjuvant treatment. In the end, 76 patients received 370 (97%) of 380 scheduled CIK cell infusion and none had WHO grade 3 or 4 adverse events. The median follow-up was of 4.4 years. The recurrence rate of HCC was significantly lower in the immunotherapy group (45%, 59 patients) than in the control group (57%, 77 patients) *p* = 0.01. The time to first recurrence was also significantly longer in the immunotherapy group than in the control group *p* = 0.008. However, the overall survival (OS) did not differ significantly between the two groups *p* = 0.09. Adoptive immunotherapy was able to lower recurrence and to extend recurrence-free time after surgery for HCC. In 2012 Xie et al. [35] published a systematic review to investigate the recurrence and survival of HCC patients after curative resection with adoptive immunotherapy. This was a meta-analysis of 6 randomized controlled trials (4 in China and 2 in Japan) including 494 patients. As adoptive immunotherapy in three trials, they used LAK cells plus interleukin-2 (IL-2), in two trials only CIKs and in one trial CIKs plus IL-2. Information over 1-year recurrence in patients was available only in two studies [36,37] with 163 patients, where recurrence in patients in the study group was significantly reduced compared to patients of the control group (OR = 0.35; 95% CI, 0.17 to 0.71; *p* = 0.003). Information over 3-year recurrence in patients was available again only for two studies [30,31] where that of patients in the study group was significantly different compared to patients of the control group (OR = 0.31; 95% CI, 0.16 to 0.61; *p* = 0.001). In the overall analysis, information over 3-year OS in patients was available only for two studies [32,33] where recurrence in patients in the study group was not significantly different compared to patients of the control group (OR = 0.91; 95% CI, 0.45 to 1.84; *p* = 0.792). The only severe side effect observed in patients receiving immunotherapy was persistent fever. In 2016 Whang et al. [38] published a systematic review investigating the recurrence and survival of patients with HCC after curative resection with adoptive immunotherapy. This was a meta-analysis of 6 randomized controlled trials including 844 patients (85.9% with hepatitis B or C). The overall analysis showed that CIK cells can improve disease-free survival DFS over the 1-year (RR = 1.23, *P* < 0.001), 2-year (RR = 1.37, *P* < 0.001) and 3-year span (RR = 1.35, *P* = 0.004). They can also improve OS over the 1-year (RR = 1.08, *P* = 0.001), 2-year (RR = 1.14, *P* < 0.001) and 3-year (RR = 1.15, *P* = 0.02) but they did not improve the 4-year and 5-year DFS and OS (*P* > 0.05). It was also found that CIK cells treatment had comparable adverse events compared to the control group (*P* = 0.39).

### 2.3. Immunotherapy with CIK Alone or in Combination with DC in Combination with TACE (Palliative)

In 2010 Hao et al. [39] published a study to investigate the efficacy of CIK cell therapy combined with TACE in patients with HCC. They did a trial, between 2005 and 2008, in which 146 patients with unresectable HCC were divided into two groups: one group (*n* = 72) was assigned CIK cell therapy in combination with TACE; the other group (*n* = 74) treated only with TACE. They analyzed the progression free survival (PFS) and OS. The PFS rates in the combination group over 6 months, 1year and 2 years were 72.2%, 40.4% and 25.3% respectively; in TACE group 34.8%, 7.7% and 2.6%. The OS rates in the combination group over 6 months, 1year and 2 years were 90.3%, 71.9% and 62.4% respectively; in TACE group 74.6%, 42.8% and 18.8%. Combined TACE with immunotherapy with CIK cells was able to improve the efficacy of TACE on HCC and to prolong the PFS and OS of HCC patients after TACE. In 2012 Wang et al. [40] published a study investigating the efficacy of CIK cell therapy combined with TACE and RFA on HCC patients. They did a trial in which 95 patients with unresectable HCC were divided into two groups: One group (*n* = 48) was assigned CIK cell therapy in combination with TACE and RFA; the other group (*n* = 47) treated only with TACE and RFA. They analyzed OS as primary endpoint and the DFS as secondary endpoint. There were 38 patients in the study group and 38 in control group who complied with the study and follow-up (44 months in median). The DFS rates in the study group over 1year, 3 years and 5 years were 79%, 26% and 16% respectively; in control group 71%, 21% and 8%. There was no significant difference between the two groups (*p* = 0.001). The OS rates in the study group over 1year, 3 years and 5 years were 92%, 53% and 26%; in control group 89%, 42% and 24%. There was no significant difference between the two groups. In 2015 Lee et al. [41] published a study where they investigated the efficacy of CIK cell therapy combined with TACE and RFA or percutaneous ethanol injection (PEI) in prolonging the recurrence free survival RFS of HCC patients. They performed a randomized, multicenter, open-label, phase 3 clinical trial, between 2008 and 2012 in hospitals in Korea, in which 230 patients who had undergone curative resection for HCC, RFA or PEI were divided into two groups: one group (*n* = 115) was assigned adjuvant cytokine-stimulated lymphocyte immunotherapy; the other group (*n* = 115) received no adjuvant treatment. They analyzed RFS as primary endpoint and OS as secondary endpoint. The median time of RFS was 44.0 months in the immunotherapy group, longer than in the control group (30.0 months). There was a significant difference between the two groups (*p* = 0.010). The OS (HR, 0.21; 95% CI, 0.06–0.75; *p* = 0.008) and cancer specific survival (HR, 0.19; 95% CI, 0.04–0.87; *p* = 0.02) was significantly longer in the immunotherapy group than in the control group. The proportion of patients who had an adverse event was significantly higher in in the immunotherapy group than in the control group (62% vs. 41%; *p* = 0.002) but the difference with serious adverse events was not significantly different between the two groups (7.8% vs. 3.5%; *p* = 0.15).

In 2016, He et al. [42] published a systematic review to investigate the efficacy of immunotherapy which includes dendritic cells and cytokine-induced killer cells (DC-CIK) in combination with TACE in HCC patients. The overall analysis showed that TACE plus DC-CIK immunotherapy can improve half-year, 1-year, and 2-year median OS and PFS in HCC patients compared to TACE alone. In 2017 Cai et al. [43] published a systematic review investigating the efficacy of immunotherapy on RFS, PFS, and OS in HCC patients in Asia. This was a meta-analysis of 12 studies (9 randomized controlled trials RCTs and 3 quasi-RCT) involving 1387 patients. Previous treatments included liver resection, TACE, RFA, percutaneous microwave coagulation therapy PMCT and PEI. The overall analysis showed that there was a significant improvement of RFS, (HR 0.56, 95% CI 0.47–0.67, *p* < 0.00001), PFS (HR 0.53, 95% CI 0.40–0.69, *p* < 0.00001) and OS (HR 0.59, 95% CI 0.46–0.77, *p* < 0.0001) in CIK group compared to control group. After CIKs therapy the proportion of CD4+ T cells increased significantly (weighted mean difference WMD 4.07, 95% CI 2.58–5.56, *p* < 0.00001), while CD8+ T cells decreased significantly (WMD −2.84, 95% CI −4.67 to −1.01, *p* = 0.002). There was no significant difference of adverse events between CIK group and control group. The clinical trials on CIK cells in HCC therapy are summarized in Table 1.

### 2.4. Gastric Cancer

Gastric cancer, is the third leading cause of cancer death among men and the fifth most common among women. In China the incidence of gastric cancer was been rising in recent years. Most cancers (about 90% to 95%) of the stomach are represented by adenocarcinomas. These kinds of cancers develop from the cells of the innermost lining of the stomach (mucosa). Before a true cancer, there are pre-cancerous changes which often occur in the mucosa of the stomach. Gastric tumors rarely cause symptoms and at time of diagnosis these patients are generally at an already advanced stage. The location of cancer in the stomach can also affect the treatment options. Long-term infection of the stomach with *Helicobacter pylori* (*H. pylori*), a bacterium colonizing the stomach, represents an important risk for distal gastric cancer. The standard treatment options for gastric cancer patients are represented by combinations of surgical resection and different chemotherapies such as platin, fluoropyrimidine, taxan and radiotherapy [44]. However, chemotherapy and radiotherapy after tumor resection only provide limited improvement in survival in patients with gastric cancer. CIKs adoptive immune therapy has been used in patients with gastric cancer in recent years. In 2015 Liu et al. [45] published a study to investigate the positive role of CIK cell therapy on gastric cancer patients in China. This was a meta-analysis of 6 case-controlled studies involving 318 patients receiving CIK cell therapy and 369 patients receiving conventional therapy. They analyzed overall OS and odds ratio (OR). The overall analysis showed a moderately increased OS in the combined group over 1-year 79 ± 10.92%, 2-year 56 ± 11.9%, 3-year 43 ± 8.31%, and 5-year 27 ± 2.44% compared to control group over 1-year 85 ± 11.3%, 2-year 69 ± 15.27%, 3-year 59 ± 12.45% and 5 year 49 ± 7.62%. These differences between the two groups were not significant but they observed a significant difference over 5-years (*p* = 0.03). The overall OR was over 1-year of 1.06 (95% CI = 0.78–1.44, *p* = 0.691), 2-year of 1.27 (95% CI = 0.88–1.81, *p* = 0.198), 3-year of 1.33 (95% CI = 0.98–1.79, *p* = 0.064) and 5-year overall of 1.77 (95% CI = 1.25–2.51, *p* = 0.001). After CIKs therapy the proportion of CD4+ T cells increased significantly, while CD8+ T cells decreased significantly in the 5-year CIK-treated group as compared to the control group with overall WMD at 15.43 (95% CI: 5.45–25.41, *p* = 0.002) for CD4+ T cell number and 0.44 (95% CI: 0.32–0.56, *p* < 0.001) for CD4+/CD8+ ratio.

### 2.5. Colorectal Liver Metastases

Colorectal cancer is one of the most common type of cancer, 2^nd^ in women and 3^rd^ in men. Adenocarcinomas represent about 96% of colorectal cancers. Risk factors for colorectal cancer include age and inflammatory bowel disease (IBD), including either ulcerative colitis or Crohn’s disease, but also a genetic/familiar predisposition. Colorectal cancer patients can eventually develop liver metastases. Colorectal liver metastases (CRMLs) patients are evaluated in 75%–85% of cases as unresectable. For these patients, systemic chemotherapy with combinations of fluoropyrimidine, oxaliplatin, irinotecan in combination with EGFR antibodies or antiangiogenic antibodies is the standard of care in this palliative situation [46,47]. Local ablative therapies can be an alternative to treat solitary metastasis. Previous studies have shown RFA as a promising treatment modality for CRMLs. It was shown that RFA can induce a tumor antigen-specific T cell response in peripheral blood which was detected by ELISPOT assay [48,49]. In 2016 Li et al. [50] published a study where they investigated the combination of CIK cell therapy and RFA for patients with CRMLs. This was a phase III, open label, non-randomized clinical trial, between 2010 and 2014, including 60 patients divided into two groups: one group RFA alone (*n* = 30); one group RFA plus CIK (*n* = 30). They analyzed PFS as primary endpoint and OS as secondary endpoint. The median PFS of the combination group and control group were respectively 23 months and 18.5 months (*P* = 0.0336). The PFS rates over 3-years were 20.3% in the combination group and 13.3% in the control group. The median OS was 43 months in control group, and not reached in combination group. The OS rates over 3-year were 81.0% in the combination group and 64.6% in control group (*P* = 0.1187). In this study 8 patients with carcinoembryonic antigen CEA >50 ng/mL were observed by ex vivo analysis (IFN-γ ELISPOT assay) for positive T cell responses against CEA-derived peptide. An increase of circulating CEA-specific T cells was found in 6 patients after RFA (*P* = 0.010), in all the 8 patients after CIK cell therapy compared to that of pre-treatment (*P* = 0.001) and in 7 patients compared to that of post-RFA (*P* = 0.028).

### 2.6. Pancreatic Cancer

Pancreatic cancer is a fatal illness usually with the identification of the illness at an already metastatic stage. Despite all therapeutic efforts, pancreatic cancer still has a very poor prognosis with a mortality rate of almost 100% [51]. Pancreatic cancer is one of the most lethal cancer pathologies, with a lower mortality rate only to the most common lung, colon and breast tumors [52,53]. The onset median is 71 years. Less than 20% of cases appear as a localized and potentially curable tumor. Despite adjuvant systemic therapies, most patients have recurrences, which means that 5 years of survival in patients is less than 5% [54]. Surgical treatment is the only potentially curative treatment of pancreatic adeno-carcinoma. Nevertheless, patients with surgery are only 15–20% and are patients in stage 1 and 2. Patients with non-resectable advanced disease or metastatic disease are generally candidates for adjuvant or neoadjuvant medical therapy. In fact, drugs today are considered the standard in locally advanced or metastatic disease, i.e., gemcitabine (GEM) and 5-fluorouracil (5-FU); both score less than 10% of responses [55]. Despite the therapeutic equivalence, gemcitabine is considered to be the standard therapeutic in the adjuvant stage also due to the better toxicity profile and the better administration. Although ductal cells represent only 20–30% of normal pancreatic parenchyma, pancreatic ductal adenocarcinoma (PDAC) is the most common pancreatic cancer with over 90% of all pancreatic cancers. The causes of pancreatic cancer are little known. Both genetic predispositions and environmental risk factors contribute to the development of pancreatic ductal adenocarcinoma. There are some inherited genetic pathologies that predispose subjects to the development of ductal adenocarcinoma of the pancreas: Peutz-Jeghers (PJS) syndrome, Lynch Syndrome, Hereditary Pancreatitis (HP), Cystic fibrosis, Hereditary breast and ovarian carcinoma (HBOC), Ataxia-teleangectasia or Louis-Bar (AT) syndrome, Familial Atypical Mole-Multiple Melanoma (FAMMM) family syndrome. Environmental risk factors and lifestyles are: tobacco, alcohol consumption, non-familial pancreatitis, diabetes, cholecystectomy, *H. pylori* infection, and hepatitis virus infection. In 2015 Chen and Zhang [56] published a study to investigate the survival of pancreatic cancer patients after curative resection with adoptive immunotherapy. This was a meta-analysis of 11 eligible clinical trials including 413 patients referred to as DC, DC-CIK, LAK, NK and GM-CSF secreting pancreatic cancer cell lines. The OS showed a significant improvement for pancreatic cancer patients who received immunotherapy compared to non-immunotherapy. The results demonstrated that the immune cytokine levels, lymphocyte subsets and serum cancer markers in the peripheral blood of pancreatic cancer patients were significantly improved after immunotherapy.

## 3. Lung Cancer

After non-melanocytic skin cancer (NMSC), lung cancer is the second most frequent type of cancer worldwide. There are two main types of lung cancer: non-small cell lung cancer (NSCLC) about 80% to 85% of lung cancers; small cell lung cancer (SCLC), called also oat cell cancer about 10% to 15% of lung cancers. Less than 5% of lung cancers are carcinoid lung tumors. Small cell and non-small cell lung cancers are treated differently. The three main types of NSCLC are: Adenocarcinoma, squamous cell carcinoma, and large cell carcinoma. A few other subtypes of NSCLC, such as adenosquamous carcinoma and sarcomatoid carcinoma, are much less common. Adenocarcinomas comprise about 40% of lung cancers. They are more common in women than in men. They grow slowly and develop from the cells which normally secrete substances such as mucus. Another type of adenocarcinoma called adenocarcinoma in situ tends to have a better outlook than other types of lung cancer. Squamous cell carcinomas are about 25% to 30% of all lung cancers. These cancers develop from squamous cells, which are flat cells. Large cell (undifferentiated) carcinomas are about 10% to 15% of lung cancers. They grow and spread quickly, e.g., large cell neuroendocrine carcinoma, very similar to small cell lung cancer. Treatment options for people with NSCLC depend on the stage and other factors. They can include: surgery, RFA, radiation therapy, chemotherapy and immunotherapy. Palliative treatments can help with symptoms. Cisplatin-based chemotherapy is the standard treatment unless high PD-L1 expression or targetable mutations are present. Immunotherapy currently has an important role in metastatic NSCLC. In 2014, Wang et al. [57] published a study investigating the efficacy of CIKs for the treatment of NSCLC. This was a systematic review including 17 randomized controlled trials of NSCLC patients including a total of 1172 patients. They were 90% metastatic or locally advanced NSCLC. The major part of patients was treated with CIK cell plus DC immunotherapy combined with chemotherapy. Patients in four of the trials were treated with CIK cells combined with chemotherapy. The median survival time MST in the CIK group was significantly prolonged compared to the non-CIK group (95% CI 27.45 to 20.66, *p* = 0.02). Three subgroups of the CIK cell group presented significant survival benefits over 1-year (OR 0.64, 95% CI 0.46–0.91, *p* = 0.01), 2-year (OR 0.36, 95% CI 0.22–0.59, *p* = 0.0001), and 3-year (OR 0.37, 95% CI 0.20–0.70, *p* = 0.002) compared to the non-CIK group. A significant difference in the long-term survival rates was found in the CIK group compared to the non-CIK group (*p* = 0.002). The median time to progression TTP was significantly prolonged in the CIK group (95% CI 22.70 to 20.47, *p* = 0.005). The results demonstrated a significant increase of CD3+ (MD 8.21, 95% CI 5.79–10.64, *p* < 0.00001), CD4+ (MD 5.59, 95% CI 4.10–7.07, *p* < 0.00001), CD3+CD56+ (MD 7.80, 95% CI 2.61–12.98, *p* = 0.003) and NK cells (CD3-CD16+CD56+) (MD 6.21, 95% CI 2.25– 10.17, *p* = 0.002) after CIK treatment, whereas the increase of CD4+CD8+ ratio (MD 2.55, 95% CI 22.46 to 7.56, *p* = 0.32) was not significantly different. In addition, the results showed a significant decreased ratio of CD8+(MD 22.75, 95% CI 23.88 to 21.63, *p* < 0.00001) and regulatory T cells (Tregs) (CD4+CD25+CD127-) (MD 21.26, 95% CI 21.94 to 20.58, *p* = 0.0003) after treatment with CIK cell therapy (Table 2). Tumor markers e.g., Ag-NORs (argyrophilic nucleolar organizer regions) and CEA in two trials [58,59] were analyzed. The analysis showed that the CIK group significantly improved Ag-NORs (MD 20.71, 95% CI 2 0.94 to 20.47, *p* = 0.00001) compared with the non-CIK therapy group. The plasma level of CEA was significantly decreased in the CIK group compared to the non-CIK group (MD 3.96, 95% CI 1.64–6.28, *p* = 0.0008). The patients in the CIK group observed fewer severe side effects from chemotherapy, such as gastrointestinal adverse reactions, anemia, grade III and IV leucopenia, and liver dysfunction compared to the group which received chemotherapy alone. The results showed that gastrointestinal adverse reactions (OR 1.77, 95% CI 1.20–2.59, *p* = 0.004) and anemia (OR 2.80, 95% CI 1.37–5.73, *p* = 0.005) generated a significant difference. Leucopenia and liver dysfunction were observed less frequently, but there wasn’t any significant difference compared with the non-CIK group. Only the incidence of fever in the CIK group was significantly higher compared to chemotherapy alone. The major part of the patients after CIK cell transfusion developed fever between 37.5 and 39 degrees, but they recovered within a few days without any severe side effects. In 2016 Mi et al. [60] published a systematic review to investigate the efficacy and safety of interleukin-2 (IL-2) and induced killer cells for NSCLC patients. This was a meta-analysis of 10 RCTs including patients with NSCLC. IL-2 has important functions in the physiological role of T-lymphocyte activation and is a growth factor [61]. It can include interferon production, cytokine production, the amplification of antibody dependent cytotoxicity and proliferative responses [62,63]. CIK cell plus chemotherapy showed a better disease control rate (OR 2.84; 95% CI: 1.35–5.97; *P* < 0.05) than chemotherapy alone. The overall analysis showed that CIK group had a significantly increased OS at the end of follow up compared to the control group with chemotherapy alone (*P* < 0.05). The hazard ratios HR (95% CI) were 0.60 (0.46, 0.79) for the postoperative treatment, 0.77 (0.60, 0.99) for combination with chemotherapy. The fixed effect model was applied to perform meta-analysis, because no significant heterogeneity was found across studies. It showed that the OS was significantly increased in the CIK cell group (HR 0.55; 95% CI: 0.35-0.87, *P* < 0.05). The random effect model showed that the OS was significantly increased in the LAK group (HR 0.54; 95% CI 0.30-0.97, *P* < 0.05). The mean differences (MDs) between the interleukin-2 or induced killer cells group and control group after treatment, were 11.32 (95% CI 6.32–16.33; *P* = 0.00001)for NK cells, 11.79 (95% CI 2.71–20.86; *P* = 0.01) for CD3+ cells, 14.63 (95% CI 2.62–26.64; *P* = 0.02) for CD4+ cells, and –4.49 (95% CI −7.80–1.18; *P* = 0.008) for CD8+ cells. Interleukin-2 and induced killer cells combination with chemotherapy showed no significant difference in ORs of anemia 0.51 (95% CI 0.14–1.93), leucopenia 0.88 (95% CI 0.47–1.63) nausea/vomiting 0.68 (95% CI 0.38–1.21), granulocytopenia 1.25 (95% CI 0.75–2.29), pulmonary toxicity 0.42 (95% CI 0.12–1.44) and diarrhea 8.20 (95% CI 0.44–153.98) compared to control group. There was a significant increase in non-infection fever ORs 6.70 (95% CI 1.44–31.13), thrombocytopenia 1.99 (95% CI 1.19–3.31) and rash (acne, pruritus) 23.40 (95% CI 2.32–235.54) (*P* < 0.05) and a significant decrease in fatigue 0.06 (95% CI 0.01–0.57) (*P* < 0.05) compared to control group. In 2018 Xiao et al. [64] published a study to investigate the efficacy and safety of the CIK cell plus radiotherapy combination in lung cancer patients. This was a meta-analysis of 16 RCTs including 1197 patients with lung cancer. CIK therapy increased the objective response rate (ORR) to 1.32, (95% CI 1.21 to 1.44), the disease control rate (DCR) to 1.13 (95% CI 1.04 to 1.23), the 1-year OS rate to 1.38 (95% CI 1.16 to 1.63) and the 2-year OS rate to 1.23 (95% CI 1.11 to 1.35). DCs-CIK cells increased the 3-year OS rate to 1.66 (95% CI 1.20 to 2.29). DCs-CIK therapy increased the CD3+T cells to 2.27 (95% CI 1.47 to 3.06), CD4+T cells to 1.28 (95% CI 0.74 to 1.81), NK cells 2.04 (95% CI 0.74 to 3.33) and CD4+/CD8+ T cells ratio 1.20 (95% CI 0.64 to 1.76) and decreased the CD8+T cells −0.84 (95% CI −1.60 to −0.08). CIK plus radiotherapy, mainly three dimensional conformal radiotherapy (3D-CRT), decreased the risk of leukopenia 0.85 (95% CI 0.76 to 0.95) and increased the risk of fever to 5.50 (95% CI 2.71 to 11.17) than that of radiotherapy alone. CIK plus radiotherapy increased the DCR, ORR, 1- and 2- year OS rate in NSCLC, but only DCR in SCLC. The systematic reviews on CIK cells in NSCLC therapy are summarized in Table 3.

## 4. Breast Cancer

### 4.1. Classification and Therapy of Breast cancer

Breast cancer (BC) occurs almost entirely in women and represents the second leading cause of cancer death among women worldwide [65], but men can get also breast cancer. Most part of BC begins in the ducts which carry milk to the nipple (ductal carcinomas), but some start in the glands which make milk of the breast (lobular carcinomas). Hereditary breast cancers represent only 5–10% of the cases of the disease [66]. This includes those who carry the BRCA1 and BRCA2 gene mutation with a hereditary breast–ovarian cancer syndrome. It is classified into 4 major molecular subtypes according to hormone receptor (HR), growth factor receptor expression, and/or extra copies of the human epidermal growth factor receptor 2 *HER2* gene (also called ERBB2): luminal A (HR+/HER2−); HER2+; luminal B (HR+/HER2+); triple negative (triple-negative breast cancer TNBC; HR−/HER2−). For each of these subtypes there are different risk factors and preferential organ sites of metastases. The nature of BC determines the therapeutic options. Luminal BCs are positive for HR [estrogen receptor (ER) and progesterone receptor (PR)]. They are about 60–80% of BC cases in developed countries [67]. Luminal A subtype grows slowly and is less aggressive than other subtypes. Luminal B subtype usually has a poorer prognosis. HER2+ BC can be treated with anti-HER2 therapies. Triple negative breast cancer is more aggressive. Endocrine therapy is the major treatment for HR+ BC. It works by lowering the hormone level or blocking the effects of hormone but metastatic HR+ BC may develop resistance to standard hormonal therapies, which was mainly due to genomic alterations. The development of new agents can reverse the resistance. Interestingly standard chemotherapy remains the treatment for TNBC with the most complete response to chemotherapy (22%). Currently available drugs are: aromatase inhibitors Als (exemestane, anastrozole and letrozole) resulting in estrogen depletion by blocking the conversion of androgens to estrogens; luteinizing hormone-releasing hormone LH-RH analogs (leuprolide and goserelin), which block the production of hormone from the ovary; tamoxifen, a prodrug that blocks the uptake of estrogen by the endoplasmic reticulum ER; fulvestrant, a selective degrade of ER which is suitable for BC patients refractory to previous hormonal therapy.

### 4.2. Meta-Analysis and Stage IV Breast Cancer with the Follow-up Time Up to 10 Years

In 2014 Wang et al. [68] published a study investigating the efficacy of CIKs alone, DCs alone and the combination of CIK and DC cells in the treatment of BC patients. This was a meta-analysis of clinical studies which included 633 patients assigned to different cohorts. The overall analysis showed that the 1-year survival of DC-CIK cells group was significantly improved (*P* = 0.0001) compared to non-DC-CIK group. The Karnofsky performance status scale can be used to compare effectiveness of different therapies. That of the patients treated with DC-CIK cells was significantly improved compared to control group (*p* < 0.0001). The overall analysis showed a significant increase in the DC-CIK treatment group of T cells CD3+, CD4+, CD4+CD8+, natural killer T cells CD3+CD56+, monocytes CD16+ (*p* ≤ 0.05) compared to non-DC-CIK group. There was no significant decrease of CD4+CD25+ regulatory T cells (*p* = 0.32). The levels of IL-2, IL-12, TNF-α, IFN-γ, and nucleolar organizer region protein were significantly increased (*p* < 0.001) after DC-CIK cell treatment. The levels of CEA, alpha-fetoprotein AFP, cancer and carbohydrate antigen CA tumor markers were significantly decreased (*p* < 0.00001) after DC-CIK treatment. In 2017 Lin et al. [69] published a study to investigate the efficacy of CIK and DC cells therapy in patients with histologically confirmed stage IV BC. This was a retrospective observational study performed between 2003 and 2013 in China including 368 patients divided into 2 groups: chemotherapy plus DC-CIK cells treatment group (*n* = 188) and control group (*n* = 180) with chemotherapy alone. Patients met the inclusion criteria if they had pathologically confirmed BC HR+ with visceral metastatic, being insensitive to endocrine therapy and had completed chemotherapy for at least 1 month. The follow-up time was up to 10 years for all patients. Patients in the DC-CIK group, after receiving low-dose chemotherapy, also received the DC-CIK therapy four times to form one cycle; at least three cycles given. All subsets of lymphocyte in terms of count were significantly decreased after chemotherapy than before chemotherapy, but they were again higher after DC-CIK immunotherapy. In terms of lymphocyte function, Th1-type cytokines (IL-2, TNF-β and IFN-γ) were decreased after chemotherapy but they were again higher after DC-CIK therapy. The most common reaction among patients was fever (34.6%). In 2017 Hu et al. [70] published a study where they investigated the efficacy and safety of DC-CIK cells therapy in BC patients. This was a meta-analysis of 11 RCTs including 941 BC patients divided into two groups: chemotherapy plus DC-CIK treatment group (*n* = 386) and control group with chemotherapy alone (*n* = 361). The complete response (CR) was reported in 9 trial including. The difference in CR was statistically significant between the DC-CIK group and control group (RR = 1.54, 95% CI: 1.09–2.19). The fixed-effect model was used. The partial response (PR) was reported in 9 trials. The difference in the PR was statistically significant (RR = 1.33, 95% CI: 1.11–1.59). The overall response rate (ORR) was reported in 10 trials. The difference in the ORR was statistically significant (RR = 1.37, 95% CI: 1.20–1.57). The incidence of leukopenia was reported in 4 trials. There was no significant difference (RR = 0.97, 95% CI: 0.86–1.09) between the DC-CIK group and control group. The incidence of thrombocytopenia was reported in 5 trials. There was no significant difference (RR = 1.29, 95% CI: 0.64–2.58) between the DC-CIK group and control group. The random-effect model was used. The systematic reviews on CIK cells in breast cancer therapy are summarized in Table 4.

## 5. Glioblastoma

Glioblastomas (GBMs) are the most common and aggressive malignant primary brain tumors in adults [71]. GBM cells can usually develop the resistance to standard treatment. The standard of treatment is represented by surgical resection followed by radiotherapy and adjuvant temozolomide (TMZ). Gene expression-based subtypes are: proneural (PN) aberrations in the platelet-derived growth factor receptor alpha (PDGFRA), mesenchymal (MES) aberrations in neurofibromatosis type I (NF1), and classical (CL) aberrations in epidermal growth factor receptor (EGFR) [72]. GBMs are highly heterogeneous also at a histological level. They have a high degree of inter- and intratumoral heterogeneity. Genetic driver mutations in GBM subtypes can create unique differences in their microenvironments. GBM cells are characterized by diffuse infiltration of the adjacent brain parenchyma. The GBM microenvironment can contain an array of non-neoplastic cells. The major part include cells of the innate immune system called tumor-associated macrophages (TAMs) constituting about 30–40% of the cells in a GBM [73]. They are infiltrating immune cell populations which are able to engage in reciprocal interactions with neoplastic tumor cells. They can promote tumor growth and progression [74]. In 2017 Kong et al. [75] published a study to investigate the efficacy and safety of CIK cell therapy combined with TMZ in GBM patients. They performed a multi-center, randomized, open-label phase III clinical trial between 2008 and 2012 in Korea. This included 180 patients divided into two groups: CIK immunotherapy group plus standard TMZ chemoradiotherapy (*n* = 91) and TMZ chemoradiotherapy alone (*n* = 89). The primary endpoint was PFS. Secondary end points were OS, objective responsive rate (ORR), DCR, Quality of life (QoL), KPS and assessment of adverse effects. There was a significant increase in the median PFS between the CIK immunotherapy group; 8.1 months (95% CI 5.8 to 8.5) compared to 5.4 months control group (95% CI 3.3 to 7.9) (*p* = 0.0401). The PFS rates with CIK immunotherapy group were 28.3% over 1 year and 18.4% over 2 years. The PFS rates with control group were 22.6% over 1 year and 13.4% over 2 years. There was a significant increase in the median OS between the CIK immunotherapy group 22.5 months (95% CI 17.2 to 23.9) compared to 16.9 months control group (95% CI, 13.9 to 21.9). However, the results did not show any statistically significant difference between the two groups. There was no significant difference in the ORR complete or partial response between the two groups (27.1% vs. 15.9%, *p* = 0.0783). However, there was a significant difference in DCR between the two groups including complete response, partial response, and no change (82.4% vs. 63.4%, *p* = 0.0058). The incidences of total and ≥ grade 3 treatment-emergent adverse events (TEAEs) were higher in the CIK immunotherapy group than the control group. However, there was no significant difference between groups. There was no significant difference in the rate of serious adverse events (41.2% vs. 36.5%, *p* = 0.5290), and in the rate of ≥ grade 3 adverse events (47.1% vs. 36.5%, *p* = 0.1616) between the two groups.

## 6. Renal Cell Carcinoma

Renal cell carcinoma (RCC) represent about 85% of kidney cancers and is more common in males than females. This cancer starts from the epithelium of the renal tubules. Risk factors of kidney cancer are: lifestyle (smoking and poor diet), environment, occupation and inherited predisposition which represent less than 4%. Three main subtypes of adult renal cancers include: Clear cell representing 70%, papillary about 10–15% and chromophobe tumors 5% [76]. Surgical resection is an effective therapy for localized RCC but stage IV or advanced disease can benefit from surgery. In 2014 Wang et al. [77] published a study investigating the efficacy of CIK cells in the treatment of RCC patients. This was meta-analysis of 7 clinical trials including 385 patients (183 controls). The overall analysis showed a significant improvement in complete and partial response between the CIK immunotherapy group compared to non-CIK therapy (*p* < 0.0001). There was a significant improvement in the OS over 1-year (*p* = 0.0002) and over 3-years *p* < 0.0001) in the CIK cell therapy group compared to non-CIK group. In 2015 Zhao et al. [78] published a study to investigate the efficacy of DC-CIK immunotherapy in different stages RCC patients. They performed a clinical trial between 2011 and 2012 in China including 122 patients. There were 60 operable patients and 62 inoperable patients. Operable patients were randomly assigned DC-CIK treatment group and control group. Inoperable patients were randomly assigned DC-CIK treatment group and inoperable control group. In 60 postoperative patients there was a significant increase in DFS in DC-CIK treatment group 96.7% over 3-year compared to control group 57.7% (*p* = 0.0418). In 62 inoperable patients there was a significant increase in OS in DC-CIK treatment group 48.8% over 3-year compared to control group 21.2% (*p* = 0.0116). The results showed an increase in the median OS in DC-CIK treatment group 28 months compared to 11 months control group. There was also a significant increase in median PFS in DC-CIK treatment group compared to control group (*p* = 0.0212). A significant increase was observed after 6 cycles of DC-CIK treatment in the rates of CD4+ T lymphocytes (*p* = 0.021) and CD4+/CD8+ (*p* = 0.002) compared to control group. It was found that DC-CIK immunotherapy was well tolerated in these patients. The general conditions of patients were significantly improved.

## 7. Hematological Diseases

The use of immune therapy for the treatment of hematological malignancies is an effective obvious treatment for hematological malignancies, as they are more accessible to effector immune cells. However, one disadvantage for these cancers is that the effector immune cells may potentially be malignant themselves. One example is myelodysplastic syndromes (MDSs) where T cells from patients may originate from the malignant clone [79]. How this can influence the effector function is yet to be clarified. Multiple myeloma (MM) is one of the most common hematological malignancies which is characterized by aberrant bone marrow plasma cells proliferation with excessive monoclonal protein production. This can eventually cause anemia or lytic bone lesions, hypercalcemia and renal failure [80]. One of the standard treatment for MM is usually able to relieve the gravity and improve survival of MM patients but most patients can relapse and develop resistance to treatment which eventually was not able to completely eradicate tumor cells. In 2017 Wang et al. [81] published a study to investigate the efficacy and safety of combination DC-CIK immunotherapy in multiple myeloma MM patients in China. This was a meta-analysis of 12 trials including 594 patients divided into two groups (as shown in Table 5): DC–CIK in combination with chemotherapy (*n* = 300) group and chemotherapy alone (*n* = 294). The overall analysis showed that there was a significant increase in CR rates, PR, ORR and DCR of DC-CIK immunotherapy with chemotherapy group compared to control group. There was a significant decrease in progressive disease PD rates and nonresponse (OR = 0.24, CI = 0.07–0.80, *p* = 0.02) of DC-CIK immunotherapy group compared to control group. There was a significant decrease in prognostic indicators including the percentage of tumor cells (OR = −35.15, CI = −43.58 to −26.71, *p* < 0.00001), levels of β2-microglobulin (OR = −13.80, CI = −17.49 to −10.10, *p* < 0.00001), M protein (OR = −17.77, CI = −21.68 to −13.87, *p* < 0.00001), 24 h urine light chains (OR = −11.39, CI = −13.00 to −9.78, *p* < 0.00001) and creatinine (OR = −215.79, CI = −246.51 to −185.06, *p* < 0.00001) of DC-CIK immunotherapy with chemotherapy group compared to control group. The results showed that there was a significant increase in CD4+ (OR = 3.14, CI = 1.65–4.64, *P* < 0.0001), CD4+/CD8+ ratio (OR = 0.38, CI = 0.18–0.59, *p* = 0.0002), cytokines levels of AgNOR (OR = 1.05, CI = 0.40–1.70, *p* = 0.002), IFN-γ (OR = 33.91, CI = 30.68–37.14, *p* < 0.00001), IL-2 (OR = 19.56, CI = 9.74–29.39, *p* < 0.0001), and IL-12 (OR = 12.25, CI = 1.53–22.97, *p* = 0.03) of DC-CIK immunotherapy with chemotherapy group compared to control group. There was a significant decrease in CD8+ (OR = −9.26, CI = −11.58 to −6.95, *p* < 0.00001), CD4+CD25+ (OR = −2.97, CI = −4.44 to −1.50, *p*<0.0001), levels of IL-4 (OR = −8.34, CI = −10.06 to −6.62, *p* < 0.00001), IL-6 (OR = −35.01, CI = −59.02 to −11.00, *p* = 0.004), IL-10 (OR = −11.10, CI = −13.13 to −9.07, *p* < 0.00001) and TGF-β (OR = −0.35, CI = −0.58 to −0.13, *p* = 0.002) of DC-CIK immunotherapy with chemotherapy group compared to control group. There were no serious adverse events or death after DC–CIK and QoL evaluated using Ps score and KPS was significantly improved (Ps: OR = −1.27, CI = −1.83 to −0.71, *p* < 0.00001; Kps: OR = 28.90, CI = 27.51–30.29, *p* < 0.00001) in DC-CIK immunotherapy with chemotherapy group compared to control group. In 2016 Peter Bader [82] performed a prospective phase I/II clinical trial including 40 patients in Germany to investigate the efficacy and safety of IL-15 activated CIK cells generated from PBMCs of the original stem cell donors in myelodysplastic syndromes and acute leukemia patients, showing evidence of relapse, who have undergone allogeneic stem cell transplantation SCT (as shown in Table 5). This study is recruiting patients and the estimated study completion date is March 2020. They will investigate the CIKs efficacy by analyzing the PFS and OS of patients over 1-year.

## 8. Future Perspectives

The immunology of tumors classically includes an area of research and therapy aimed, on the one hand, to investigate the interactions between the immune system and cancer and, on the other, to identify and propose therapeutic solutions based on the stimulation of the effector components of the immune system. The field of immunotherapy applied to the treatment of tumors is experiencing a moment of great transformation. There are several therapeutic approaches, but certainly one of the most fascinating is based on the use of effector cells of the immune system (cellular immunotherapy), armed selectively against cancer cells. Adoptive cellular immunotherapy (ACI) is based on the administration of T lymphocytes recognizing and targeting selectively specific tumor cells. It is a therapeutic tool that has proven to be effective on some types of cancer. The specificity of recognition is conferred by TCR—T lymphocyte receptor. Based on the characteristics of TCR, the lymphocyte can recognize, for example, tumor antigens and therefore attack the tumor cells provided with that particular antigen. Immunotherapy is certainly a promising emerging strategy, with an almost non-toxic profile. The last frontier of immunotherapy is that in which the T lymphocytes are modified to express a synthetic protein, CAR or chimeric antigen receptor in which an antibody that recognizes an antigen expressed by tumor cells is bound to molecules that activate T cells. This way CAR-T cells can recognize tumor cells through the antibody and activate a series of mechanisms that ultimately lead to killing cancer cells. It started from the oncological diseases of the blood to get, even step by step, to solid tumors [83,84]. CAR-T is a complex therapy which is diverse and potentially serious side effects are also associated, such as cytokine release syndrome, a massive release of molecules implicated in inflammatory processes.

Cytokine Induced Killer cells, T cells expanded in vitro and characterized by a potent anti-tumor activity, represent an encouraging immunotherapy strategy in the oncological field. Oncological immunotherapy is authorized in Europe, in line with the most advanced studies that show how this type of approach can concretely improve the life of some patients, even in the case of childhood cancers. During the early stage of cancer, the immune system has the ability of identifying and eliminating cancer cells in the process of immuno-editing in which cancer cells could escape from a balanced status of equilibrium by the immune system. Usually cancer cells are able to suppress antitumor responses by secreting immunosuppressive factors. These interfere with dendritic cell maturation and function in the lymph nodes which lead to an inefficient tumor response. DCs are indispensable in presenting antigens and secreting cytokines. The use of antigen-pulsed autologous dendritic cells to stimulate cytotoxic T-cell anti-tumor immune responses has been developed in clinical trials in humans with cancer.

## 9. Conclusions

Immunotherapy is a promising treatment for a number of cancers. CIK cells have a significant antitumor activity and are able to eradicate tumors with few side effects. The differences of the outcomes in different kinds of cancers could result from different molecular mechanisms unexplained yet. The majority of studies were performed in Asia leading to a regional limitation. In 2015, the report of results from International Registry on CIK Cells (IRCC) was published [85]. In order to have defined appropriate research protocols it is important to standardize the application of combination therapy to have more exhaustive information of patient follow-up.

## Figures and Tables

**Table 1:** Clinical studies applying CIK cells for the treatment of HCC.

Study Reference	Patients (*n*) Total	Patients (*n*) Treated with CIK Cells	Therapy	Results	Conclusions
Takayama et al. (2000) [34]	150	76	Resection; Immunotherapy group: additional infusions of lymphocytes activated in vitro with rIL-2 and anti-CD3	Better recurrence-free in immunotherapy group; no differences in OS between the two groups	CIK cell therapy can improve recurrence-free outcomes after surgery
Hao et al. (2010) [39]	146	72	TACE; Immunotherapy group: additional i.v. CIK cell transfusions	Improved PFS and OS in TACE plus CIK cell therapy group	Adjuvant immunotherapy with CIK cells improve the efficacy of TACE in HCC patients
Wang et al. (2012) [40]	95	48	TACE/RFA; Immunotherapy group: additional CIK cell transfusions	Significantly longer DFS in CIK cell plus TACE with RFA group; no significant difference for OS	Recurrence can be controlled by CIK infusion combined with TACE and RFA
Lee et al. (2015) [41]	230	115	TACE/RFA or percutaneous ethanol injection PEI Immunotherapy group: additional i.v. CIK cell transfusions	RFS and OS significantly better in immunotherapy group; no significant for severe adverse events between the two groups	CIK cells can prevent recurrence and improve survival in combination with surgical resection, RFA or percutaneous ethanol injection

**Table 2:** Lymphocytes subsets in peripheral blood before CIK treatment and after CIK therapy (modified from [57]).

Event	No. of Trials	No. pts	Before-CIK	Mean Difference	95% CI	*p* Value	Heterogeneity
CD3+	9	359	359	8.21	5.79 to 10.64	<0.00001	67%
CD4+	5	174	174	5.59	4.10 to 7.07	<0.0001	0%
CD3+CD8+	4	174	174	2.55	−2.46 to 7.56	0.32	89%
CD4+CD8+	4	144	144	0.49	0.37 to 0.61	<0.00001	53%
CD3+CD56+	6	222	222	7.80	2.61 to 12.98	0.003	99%
NK	4	154	154	6.21	2.25 to 10.17	0.002	90%
CD8+	5	174	174	-2.75	−3.88 to −1.63	<0.00001	0%
Treg	3	153	153	-1.26	−1.94 to −0.58	0.0003	58%

**Table 3:** Systematic reviews applying CIK cell and DC cell for the treatment of NSCLC.

Study Reference	RCTs (*n*) Total	Patients (*n*) Total	Therapy
Wang et al. (2014) [57]	17	1172	Chemotherapy; Immunotherapy group: additional CIK cell and DC cell transfusions
Mi et al. (2016) [60]	10		Chemotherapy; Immunotherapy group: additional CIK cell transfusions and IL-2
Xiao et al. (2018) [64]	16	1197	Radiotherapy; Immunotherapy group: additional CIK cell and DC cell transfusions
Total	43	2369	

**Table 4:** Systematic reviews applying CIK cell and DC cell for the treatment of Breast cancer.

Study Reference	RCTs (*n*) Total	Patients (*n*) Total	Therapy
Wang et al. (2014) [68]		633	CIK cell therapy alone, DC cell therapy alone and CIK and DC combination
Hu et al. (2017) [70]	11	914	Chemotherapy; Immunotherapy group: additional CIK cell and DC cell transfusions
Total	11	1547	

**Table 5:** Clinical studies applying CIK cells for the treatment of hematological diseases.

Study Reference	RCTs (*n*) Total	Patients (*n*) Total	Therapy	CR	PR	ORR	DCR	PD
Wang et al., 2017, [81]	12	594	Chemotherapy;	OR = 2.71 CI = 1.60–4.58 *p* = 0.0002	OR = 1.49 CI = 1.01–2.20 *p* = 0.04	OR = 2.77 CI = 1.88–4.10 *p* < 0.00001	OR = 2.90 CI = 1.72–4.90 *p* < 0.0001	OR = 0.34 CI = 0.20–0.58 *p* < 0.0001
			Immunotherapy group: additional CIK cell and DC cell transfusions					
Bader, 2016, [82]	1	40	Chemotherapy;	n.a	n.a	n.a.	n.a.	n.a.
			Immunotherapy group: additional IL-15 activated CIK cell transfusions					
Total	13	634						

n.a., not available; OR = odd ratio; CI = coefficient of interference.
